# Growth and Optical Properties of the Whole System of Li(Mn_1-*x*_,Ni*_x_*)PO_4_ (0 ≤ x ≤ 0.5) Single Crystals

**DOI:** 10.3390/ma14237233

**Published:** 2021-11-26

**Authors:** Benyan Xu, Zhenyou Li, Kunpeng Wang, Jianxiu Zhang, Lanju Liang, Longfei Li, Yanbiao Ren, Yong Liu, Meng Liu, Dongfeng Xue

**Affiliations:** 1 Laboratory of Crystal Materials, Zaozhuang University, Zaozhuang 277160, China; byxud@uzz.edu.cn (B.X.); zjjdjxk@uzz.edu.cn (J.Z.); lajdkfj@uzz.edu.cn (L.L.); ybrendk@uzz.edu.cn (Y.R.); Ylifjlk@163.com (Y.L.); mloijd@uzz.edu.cn (M.L.); 2 Electrochemical Energy Storage, Helmholtz Institute Ulm (HIU), Helmholtzstraße 11, D-89081 Ulm, Germany; zjdlfu@uzz.edu.cn; 3 State Key Laboratory of Advanced Metals and Materials, University of Science and Technology Beijing, Beijing 100083, China; Longfeijf@163.com; 4 Multiscale Crystal Materials Research Center, Shenzhen Institute of Advanced Technology, Chinese Academy of Sciences, Shenzhen 518055, China

**Keywords:** LiMPO_4_ (M = Fe; Co; Ni; Mn), single crystal growth, crystal characterization, floating zone method

## Abstract

A series of single crystals of Li(Mn_1-*x*_,Ni*_x_*)PO_4_ (*x* = 0.00, 0.01, 0.02, 0.03, 0.04, 0.05, 0.06, 0.08, 0.10, 0.15, 0.20, and 0.50) have been grown to large sizes up to 5 mm in diameter and 120 mm in length using the floating zone method for the first time. The comprehensive characterizations of the as-grown crystals were performed before further physical property measurements. The composition of the grown crystals was determined by energy-dispersive X-ray spectroscopy. The crystal structures were characterized by the X-ray powder diffraction method with a GSAS fitting for structural refinement, which reveals a high phase purity of the as-obtained crystals. The polarized microscopic images and Laue patterns prove the excellent quality of the single crystals. Oriented cuboids with sizes of 2.7 × 3.8 × 2.1 mm^3^ along the *a*, *b*, and *c* crystalline directions were cut and polished for further anisotropic magnetic and transparent measurements. We also first proposed a new potential application in the non-linear optical (NLO) and laser generation application for LiMPO_4_ (M = transition metal) materials. The optical and laser properties, such as the absorption spectra and the second harmonic generation (SHG), have been investigated and have furthermore confirmed the good quality of the as-grown single crystals.

## 1. Introduction

Lithium transition metal phosphates LiMPO_4_ (M = Fe, Co, Ni, Mn) with an olivine structure are multifunctional materials with great application prospects and have become one of the research hotspots for rechargeable batteries and high-security data storage fields. Especially in the field of energy storage technologies, Prof. John B. Goodenough, the father of lithium-ion batteries, discovered in 1997 [1] that LiFePO_4_ is a potential cathode material with high safety, a low cost, and a long lifespan, which, therefore, opened the door to the practical application of the lithium-ion batteries. Due to this contribution, Prof. Goodenough was awarded the 2019 Nobel Prize in Chemistry [2]. In addition, in 2020, Huawei has officially decided to use the LiFePO_4_ lithium batteries as the power supplier for their huge data centers and broadly distributed telecom base stations, including the future 5G [3]. Just as the Nobel’s official comment to Prof. Goodenough’s work: They have laid the foundation of a wireless, fossil-fuel-free society and are of the greatest benefit to humankind [4].

Although LiFePO_4_ is currently one of the dominant cathode materials in the power battery industry, its practical average energy density is still as low as ca. 140 Wh/kg, which is far from reaching its theoretical value of 180 Wh/kg [5]. For more than twenty years, many efforts have been made all over the world to investigate the lithium exchange principles in LiFePO_4_ and to optimize its properties by doping the transition metal ions, such as Ni, Co, and Mn, etc. [6]. It has been demonstrated that mass transport in such an olivine structure suffers from an isotropic nature, leading to a poor rate capability of LiFePO_4_ cathodes. [7] The anisotropic Li-ion diffusion is also the main reason for the electrochemical performance deviation of LiFePO_4,_ even synthesized under the same experimental conditions, due to different crystal faces exposed in the polycrystalline products [8,9,10,11]. In this regard, the single crystals of the LiMPO_4_ family have certain advantages over their polycrystalline powders to investigate the anisotropic nature of the lithium exchange and electron transfer in this kind of material [12,13,14].

Understanding the intrinsic property of LiMPO_4_ is also very important to other application fields, such as data storage. Prof. Manfred Fiebig from Switzerland first discovered a novel ferroic order—ferrotoroidicity (the other three being the well-known ferromagnetism, ferroelectricity, and ferroelasticity)—in LiCoPO_4_ polycrystals [15,16,17,18]. This interesting fourth type of ferroic order opened a new chapter for the study of multiferroic materials, and furthermore, this magnetic ordered state can be used to make new and very stable data storage devices [19]. In order to thoroughly investigate the intrinsic nature, especially the anisotropic properties along different axes of this new ferrotoroidicity and the formation mechanism of the multi-domain structure, the well-studied polycrystalline system must be replaced by the high-quality single crystals [20,21,22,23]. 

In this paper, we have grown a first series of high-quality single crystals of Ni-doped Li(Mn_1-*x*_,Ni*_x_*)PO_4_ (*x* = 0 ∼ 0.5) of a large size, up to 5 mm (D) × 120 mm (L). The X-ray powder diffraction (XRD) with GSAS fitting was applied to characterize the crystal phase of the obtained single crystals and determine the fine structure. The polarized images and Laue patterns prove their excellent optical quality. The Laue backscattering is a powerful technique to identify the orientation and find out the symmetry of the projection for a single crystal. Oriented cuboids along the *a*, *b*, and *c* crystalline directions were cut and polished for further anisotropic magnetic and transparent measurements. Furthermore, a new application on non-linear optical (NLO) and laser generation has been proposed. 

## 2. Experimental Section

### 2.1. Crystal Growth

Polycrystalline samples of Li(Mn_1-*x*_,Ni*_x_*)PO_4_ with *x* = 0.00, 0.01, 0.02, 0.03, 0.04, 0.05, 0.06, 0.08, 0.10, 0.15, 0.20, and 0.50 were prepared by using solid-state reaction techniques with the stoichiometric mixture of Li_2_CO_3_ (Chempur 99+%, Karlsruhe, Germany), MnCO_3_ (Aldrich 99.9+%, Beijing China), NiO (Aldrich 99.9+%, Beijing China), and NH_4_H_2_PO_4_ (Chempur 99+%, Karlsruhe, Germany) under Argon flowing, respectively. The chemical equation can be expressed as follows [24]:

$${\mathrm{Li}}_{2}{\mathrm{CO}}_{3}+2(1-x){\mathrm{MnCO}}_{3}+2x\mathrm{NiO}+2{\mathrm{NH}}_{4}H_{2}{\mathrm{PO}}_{4}\to 2\mathrm{Li}({\mathrm{Mn}}_{\left[1-x\right]},{\mathrm{Ni}}_{\left[x\right]}){\mathrm{PO}}_{4}{+\mathrm{CO}}_{2}{+H}_{2}{O+\mathrm{NH}}_{3}$$

The mixtures were ground carefully to ensure homogeneous mixing and then were sintered for 20 h at 800 °C. Consequentially, the products with different Ni-doped concentrations were pressed to polycrystalline feed rods (EPSI Engineered Pressure Systems; 2000 bar, Karlsruhe, Germany) in latex tubes with diameters of 6–8 mm and sintered again at 800 °C for 10 h.

All crystals were grown in a floating zone facility with optical heating. In order to meet the wavelength of quantum dependence, the lights of four 300 W air-cooled xenon lamps were reflected and focused in the crystal growth chamber. A quartz tube with 2 mm wall thickness was used as the growth chamber. The applied Ar pressure in the growth chamber was 2 bar. The feed rod was rotated clockwise at a rate of 15 rpm, and the seed rod anticlockwise at 15 rpm. The growth rate was 2.5–5.0 mm/h. 

A number of floating-zone experiments with different Ni-doping concentrations ranging from 0 to 0.5 have been conducted in order to further the investigation of the effect on the crystal by doping, perfecting the improvement of the grown crystals.

### 2.2. Crystal Characterization

The element contents of grown crystals were determined by electron probe microanalysis, applying the energy-dispersive X-ray (EDX) mode, which is local and non-destructive. To minimize the measurement uncertainty, we measured three different points (µm-sized) and took the root mean square deviation as the measurement error. The phase purities were checked by X-ray powder diffraction with a Rigaku X-ray diffractometer (Beijing, China) using the Cu-Ka radiation [25]. The X-ray diffraction data were analyzed using the program GSAS. The microstructures and crystal perfection of samples were investigated by optical microscopy in a polarization microscope Axiovert 25 (Gottingen, Germany) equipped with a Carl Zeiss digital camera. The orientation of the single crystals was determined by the X-ray Laue backscattering method. 

## 3. Results and Discussions

### 3.1. Crystal Growth

**Figure 1:**
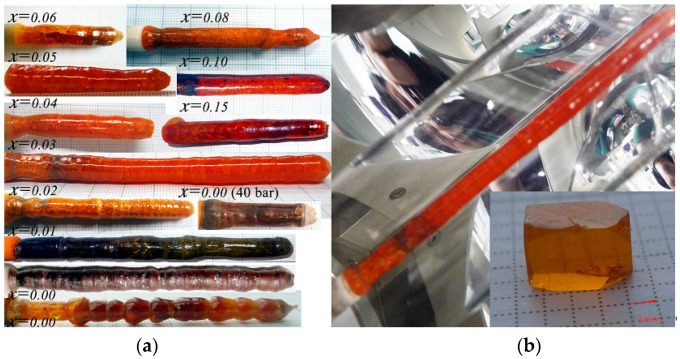
(**a**) As-grown crystals of Li(Mn_1-*x*_,Ni*_x_*)PO_4_ with different Ni-doping concentrations, and among them the size of Li(Mn_0.97_,Ni_0.03_)PO_4_ is up to 5 mm (D) × 120 mm (L); (**b**) As-grown Li(Mn_0.97_,Ni_0.03_)PO_4_ single crystal and the samples cut from it.

Figure 1a shows the as-grown crystals of Li(Mn_1-*x*_,Ni*_x_*)PO_4_ with different Ni-doping concentrations. Among them, the size of Li(Mn_0.97_,Ni_0.03_)PO_4_ is up to 5 mm in diameter and 120 mm in length. To the best of our knowledge, it is the largest LiMPO_4_ (M = Fe, Co, Ni, Mn) crystal that has been reported at present. From Figure 1a, we can see that the grown crystals display green, black, dark- or bright-orange transparent colors, depending on the different growth conditions and Ni-doping concentrations. For 0.02 ≤ *x* ≤ 0.5, the color of the crystals is basically orange, which becomes darker with an increase in the doping amount. When *x* > 0.10, a black color appears in some local parts of the crystals, indicating that the valence of Mn or Ni ions might have been changed during the crystal growth. When *x* < 0.02, the grown crystals usually display dark green, black, or brown colors, which is different from what was previously reported. To investigate the color display mechanisms, we also grew the undoped LiMnPO_4_ by using the newly designed Floating Zone facility at IFW Dresden under the same growth conditions, such as the high Ar pressure up to 40 bar, reported in the former literature [26]. However, the obtained crystal displays pale brown rather than the reported bright orange, as shown in Figure 1a, indicating that the color of the crystal depends on not only the growth conditions but probably also on the feed rod fabrication process. From this view, we made various feed rods with different sintering conditions and found that the grown crystals display the same orange color that was previously reported when decreasing the sintering time of the feed rods. Moreover, we also found that the color of the crystal could be affected by the growth rate. From Figure 1a, we can see that for the crystal with *x* = 0.01, the color was changed from black (growth rate of 2 mm/h in the starting phase) to dark green (5 mm/h when the growth process had lasted ∼10 h). In general, the valence of the metal ions is the direct influential factor on the color of a crystal. In order to prevent the oxidation or reduction of the Mn^2+^ and Ni^2+^ ions in Li(Mn_1-*x*_,Ni*_x_*)PO_4_, the control of the sintering atmosphere, temperature, pressure, time of the feed rod, and growth atmosphere is very important. In addition, we suggested that the crystal color could be changed by annealing because the stress in crystals could be released, while the valance of Mn and Ni, as well as the Li content, might also be changed. 

The stability of the crystal growth of Li(Mn_1-*x*_,Ni*_x_*)PO_4_ is greatly affected by the viscosity and homogeneity of the melt in the molten zone, which vary as the Ni-doping concentration changes. For Li(Mn_1-*x*_,Ni*_x_*)PO_4_ crystals with a doping amount *x* < 0.02, even though the homogeneity of the melt is good, the melt is likely to overflow from the molten zone because of the low viscosity, therefore leading to the difficult control of the crystal growth with equal diameters. For the doping concentrations from *x* = 0.02 to *x* = 0.15, the crystal growth becomes more stable due to the good homogeneity and suitable viscosity of the melt. When increasing the doping concentrations to *x* > 0.15, the stability of crystal growth became worse, which was induced by the non-homogeneity of the melt. In this case, gray-white precipitated phases with a high melting point were observed in the melt or attached on the surface of the melt, which has been identified as the Ni metal by X-ray powder diffractions. The precipitated Ni metals hindered the convection flow of the melt, cut off the melt, and even interrupted the growth process.

### 3.2. Crystal Characterization

To make sure we have grown single crystals rather than polycrystals by the floating zone method, a full characterization is always essential before the further investigation of the physical properties.

**Figure 2:**
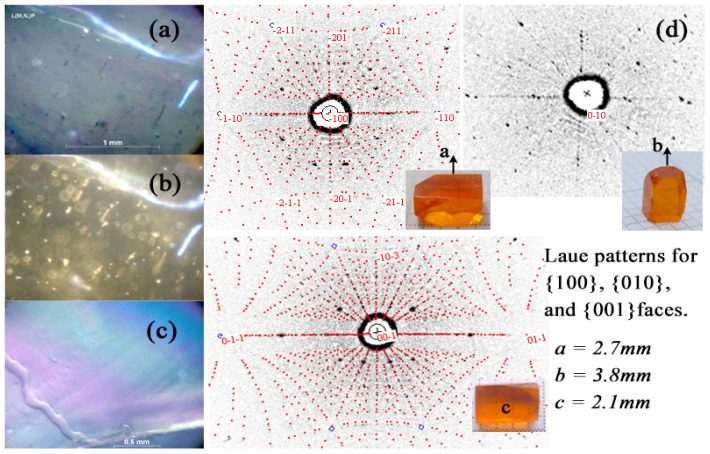
Polarization images in polarized light of both the (**a**) Q5, and (**b**) Q6 cross-sections cut from Table 0. The Ni_0.03_)PO_4_ crystal, as well as the polarization images of (**c**) a 2 mm plate cut from Li(Mn_0.97_,Ni_0.03_)PO_4_ crystal boule; (**d**) The X-ray Laue backscattering images along the *a*, *b*, and *c* axes.

The different local defects in Li(Mn_1-*x*_,Ni*_x_*)PO_4_ crystals were probably induced by crystallization front instability and their evolution during the growth process could be observed by a polarization microscope. A 5 mm thick sample was cut from the initial part of the Li(Mn_0.97_,Ni_0.03_)PO_4_ crystal boule (Figure 1b). The surface close to the initial part is labeled as Q5, and the other side is labeled as Q6. The optical images of both sides in polarized light are shown in Figure 2a,b, respectively. From both the figures, we can see that the transmittance of the polarized light is basically homogeneous, indicating that the as-grown crystal boule is single crystalline throughout the entire cross-section. The few observed high transmission areas on both surfaces were induced by the local stress, which can be eliminated by thermal annealing. Besides the local stress, we also observed a few mechanical scratches and grain boundaries. No big difference can be observed on both sides of the sample, indicating that the growth conditions of the crystal, such as the applied power, growth rate and speed of rotation, were relatively stable. The preliminary assessment shows that the as-grown crystal quality is good because the common macro-defects such as cracks, precipitations, inclusions, striations and bubbles were not observed. To further investigate the defects in the grown crystal, we cut the samples with a thickness lower than 2 mm (Figure 1b) from a Li(Mn_0.97_,Ni_0.03_)PO_4_ crystal boule. The polarized microscopic image is shown in Figure 2c. The regular interference fringe can be obviously observed, implying that the optical homogeneity of the crystal is good.

For further anisotropic physical property measurements, an orientation of the samples is essential. Generally, the distinctive facets are helpful to the crystal orientation and quality assessment, but unfortunately, our grown crystals did not show the distinctive facets, which made the orientation of the crystals very challenging. The X-ray Laue backscattering technique is a powerful tool for crystal orientation, quality assessment, and confirmation of whether the FZ grown material is a single crystal, polycrystalline, or non-crystalline. In our orientation experiment, an oriented cuboid shown in Figure 1b with dimensions of 2.7 × 3.8 × 2.1 mm^3^ along the *a*, *b*, and *c* crystalline directions was cut from the above-mentioned cylindrical sample. Laue diffraction experiments were performed; the power was 50 kW with a current of 40 mA, and the explosion time on Fuji type-80 film was 30 min. The white-radiation topography camera of the station allows the specimen to be rotated around the axes. By using such a camera, one can rotate an IQC grain to any desired orientation parallel to the incident beam. 

The X-ray Laue backscattering pattern of the 5 mm thick cylindrical sample is shown in Figure 2d. The Laue spots can be clearly observed on the film, and the identical shape of the spots indicates that the sample plan was parallel to the film. The regular and fully separated spots confirmed that there were no twins, stacking faults, interstitial stacking layers, and obvious deformation of the lattice planes in the sample. Seven diffractions with the highest intensities were selected to simulate the geometric distribution of the Laue spots based on the orthorhombic lattice unit of LiMnPO_4_. The simulated Laue patterns were in good agreement with the experimental ones, and they also demonstrated the single-crystalline nature of the sample. All the diffraction spots were then indexed successfully, confirming that the structure of the sample was basically the same with LiMnPO_4_. The Laue spot located in the center of the film was indexed as (21ī), which corresponds to the lattice plane parallel to the film. As the sample was cross-cut from the Li(Mn_0.97_,Ni_0.03_)PO_4_ crystal boule, we can deduce that the crystal was grown along the <21ī> crystalline directions. The next step was to orient the sample and cut the cuboids along the *a*, *b*, and *c* crystalline directions. Firstly, we moved the (100) diffraction spot to the center of the Laue pattern by rotating the crystal sample that was fixed on a universal goniometer. Then, we cut the crystal sample parallel to the film, polished the new cut surface, and performed the Laue experiment again. The indexing results showed that the spot located in the center of the film was diffracted from the (100) planes. Until now, we have cut the (100) faces successfully. In the same way, the (010) and (001) faces were also oriented and cut by rotating the goniometer. The final oriented cuboids along the *a*, *b*, and *c* crystalline directions are shown in Figure 1b.

**Figure 3:**
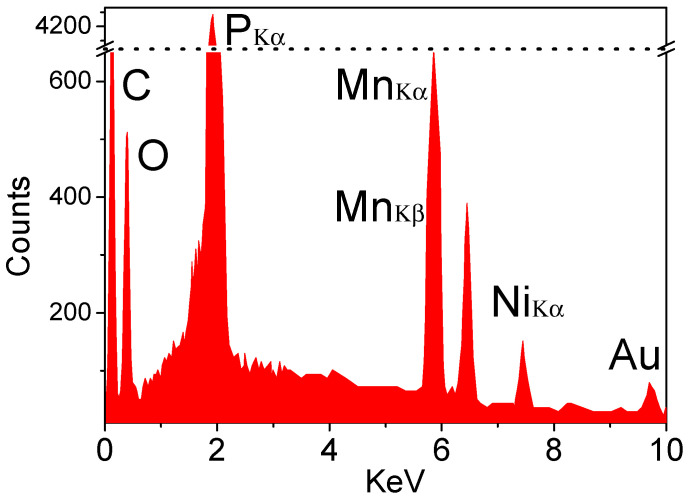
The typical EDX spectrum of the Li(Mn_0.97_,Ni_0.03_)PO_4_ crystal.

A chemical analysis is an essential experiment for the characterization of FZ-grown crystals. Note that the content of the light element Li cannot be accurately determined by microprobe analysis, neither in the EDX nor in the WDX mode. However, for our newly grown Li(Mn_1-*x*_,Ni*_x_*)PO_4_ crystals with different Ni-doping concentrations, more attention should be paid to the contents of Mn and Ni in the crystals than to that of Li, O, or P elements. Although WDX has a better spectral resolution, EDX was used in our measurements because of the short measurement time and to avoid applying the standard materials. We used the above oriented cuboids as the measurement sample. As the EDX mode is a local measurement, three different spots (µm-sized) on the (001) surface of the sample were selected to determine the chemical composition. The typical EDX spectrum is shown in Figure 3, and we can see that besides Mn, Ni, P, and O, the peaks of C and Au also appear. Here, we only focus on the contents of Mn and Ni. The measurement data on the three spots are listed in Table 1. 

From Table 1, we can see that the composition of the crystal (48.56% P, 48.04% Mn and 3.40 Ni in At%) is close to the nominal Li(Mn_0.97_Ni_0.03_)PO_4_ stoichiometry (50% P, 47.5% Mn and 2.5% Ni). The concentration values 5.90 at%, 6.92 at%, and 7.03 at% Ni at the three measurement spots, respectively, confirmed both the homogeneity and the slight Ni excess with Mn/Ni = 0.97/0.03 = 32 on the (001) surface of the crystal. To minimize the measurement error, the average values of the three detected spots, i.e., 6.62 at% Ni and 93.38 at% Mn, were taken, resulting in a Mn/Ni ratio of 14.1, which is in good agreement with the initial weight. Overall, we did not find a large deviation of the measured Ni concentrations from the initial weight, indicating that all the initially weighed Ni was incorporated in the main phase. 

### 3.3. XRD and Structures

**Figure 4:**
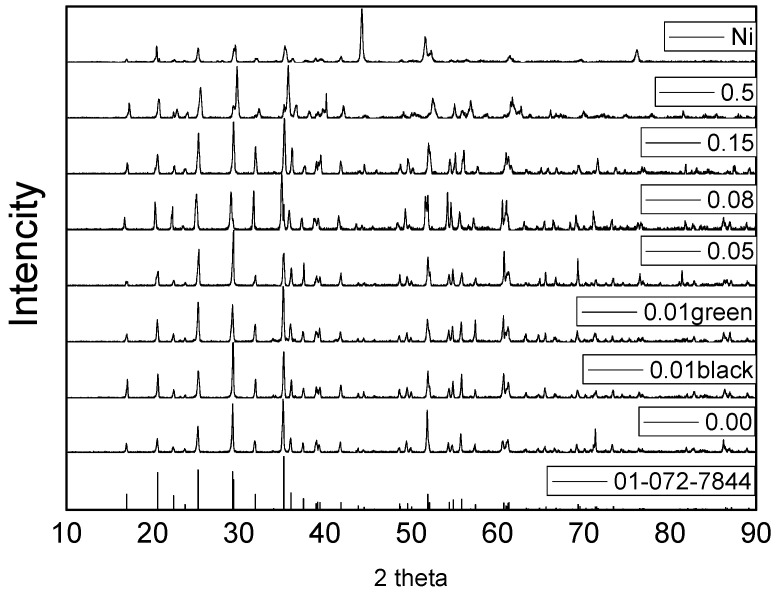
X-ray powder diffraction patterns for Li(Mn_1-*x*_,Ni*_x_*)PO_4_.

The phase purity and lattice parameters of the grown crystals were characterized by XRD diffraction. Figure 4 shows the XRD patterns for all the grown crystals, and we can see that the low Ni-doping concentrations such as *x* = 0.00, 0.01, 0.02, 0.03, 0.04, 0.05, 0.06, 0.08, 0.10, and 0.15 were found to be single-phase with the olivine-like structure. However, the samples with high Ni-doping concentrations *x* = 0.2 and *x* = 0.5 were found to be multiple phases. Samples with *x* = 0 (under 2 and 40 bar) and x = 0.01 for the black color with a growth rate of 2 mm/h are in good agreement with the LiMnPO_4_ pure phase (072-7844). Samples with *x* = 0.05 and 0.08 match well with Li(Fe_0.06_Mn_0.94_)PO_4_ (01-073-7353). The XRD patterns for *x* = 0.15 match Li(Fe_0.27_Mn_0.83_)PO_4_ (01-073-7354). Samples with *x* = 0.01 for the green color with a growth rate of 3.8mm/h matches LiMnPO_4_ (main phase), and the impurity phase of Li(Mn, Ni)PO_4_ can be observed. For *x* = 0.5, the main phase was Li(Fe_0.89_Mn_0.11_)PO_4_, and the impurity phases are Li(Fe_0.06_Mn_0.94_)PO_4_, Li(Fe_0.5_Mn_0.5_)PO_4_, and Ni metal (attached on the surface of the crystal).

**Figure 5:**
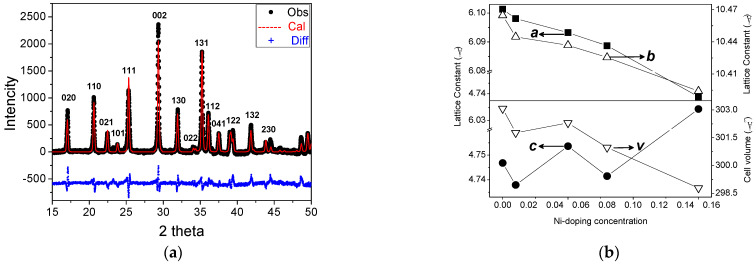
(**a**) Results of fitting the X-ray powder diffraction data of Li(Mn_0.97_Ni_0.03_)PO_4_: experimental (points), calculated (solid line), and difference (bottom); (**b**) Lattice parameters and unit-cell volumes of Li(Mn_1-*x*_,Ni*_x_*)PO_4_ with different Ni-doping concentrations.

The crystal structures of the grown crystals were refined by the GSAS program v2.1. As an example, we show the result of fitting the experimental X-ray diffraction pattern for Li(Mn_0.97_Ni_0.03_)PO_4_ in Figure 5a using the revised structure of Li(Fe_0.06_Mn_0.94_)PO_4_ as the starting model. The difference between the observed and calculated data is shown, and all the reflections are indexed. An excellent agreement is achieved, and no foreign peaks can be found. The patterns corresponding to the other Ni-doping Li(Mn_1-*x*_,Ni*_x_*)PO_4_ crystals are similar.

The derived lattice parameters of the grown crystals by XRD patterns are shown in Figure 5b, and we can see that the unit-cell parameters for the pure LMP are in good agreement with the previously reported data. The *a* and *b* parameters decrease with an increasing Ni concentration. The *c* parameter decreases with an increasing Ni concentration when *x* < 0.05; however, it increases when the doping concentration is higher than 0.05. The space groups of the crystals transform from P_nmb_ to P_nma_, and the *a* and *b* axes are swapped when the doping concentration is higher than 0.15. The parameter *a* lies between 6.07 and 6.11 Å, *b* lies between 10.37 and 10.48 Å, and *c* varies from 4.72 to 4.75 Å. The overall variation of the unit-cell volume is approximately 1.43%.

### 3.4. Linear Optical Properties

**Figure 6:**
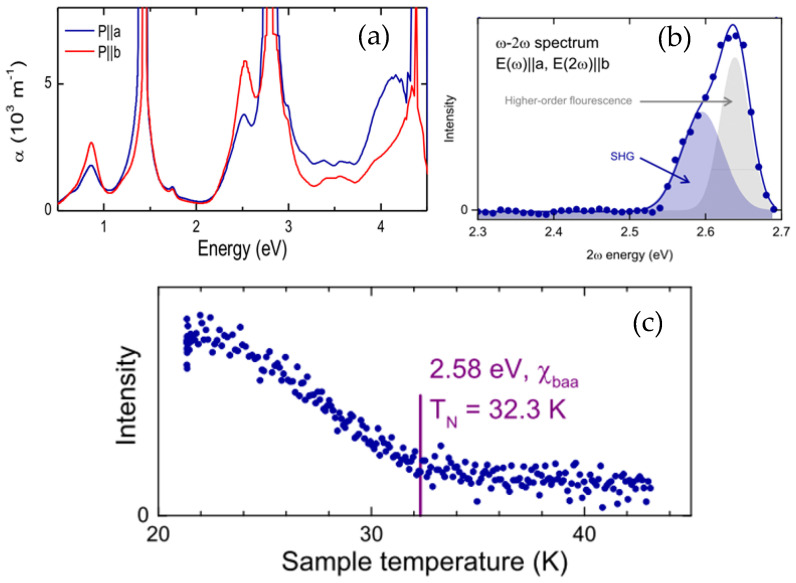
(**a**) The linear spectroscopy with a light incident along c shows strong absorption in the visible and infrared regions. Two strong absorption peaks are observed as 1.4 and 2.7 eV, corresponding to the *d*^8^ orbital of Ni^2+^ and *d*^5^ of Mn^2+^ ions, respectively, (**b**) the nonlinear excitation spectra with a light incident along c direction, (**c**) the lower-energy SHG signal.

The linear absorption spectra have been measured on the Ni-doped LiMnPO_4_ single crystals (3%) along different crystallizations. Figure 6a shows the polarized linear spectroscopy with a light incident along *c*, and we can see the strong absorption in the visible and infrared regions. We calculated the linear absorption coefficient from the measured optical density O.D. via *a* = In(10)O.D./*d*, with the crystal thickness *d* = 1.2 mm. The calculated value of *a* is a little overestimated due to scattering losses on the unpolished surfaces. The peak at 1.4 eV most probably comes from the Ni^2+^ (*d*^8^) doping, while the transitions around 2.4 eV to 3 eV can be related to the Mn^2+^ (*d*^5^) ions.

The spectrum above 4 eV is not correct, as the sample was placed on a glass slide (which does not transmit in the UV). For the undoped LiMnPO_4,_ the band gap is around 4.5 eV. 

### 3.5. Nonlinear Optical Properties

The non-linear *ω* − 2*ω* spectrum was obtained by tuning the incoming photon energy *ħω* while simultaneously detecting the signal at 2*ħω*. For the analysis, the peaks were fitted with a multi-Gauss function. Figure 6b shows the Non-linear excitation spectra with a light incident along *c* directions, and we revealed a two-peak structure around 2.6 eV. The higher-energy peak is related to a higher-order fluorescence and will probably not occur if the system is observed with a *ns* laser system (here, a *fs* laser was used).

### 3.6. The Second Harmonic Generation (SHG) Laser Generation

The second harmonic generation (SHG) experiments have been measured, and the lower-energy SHG signal shown in Figure 6c shows a decrease in temperature and vanishes around 32 K, close to the Neel Temperature T_N_ = 32.5 K of the undoped LiMnPO_4_.

The lower-energy peak corresponds to a second harmonic (SHG) response and couples to the symmetry-breaking magnetic order parameter. The latter is demonstrated in the temperature-dependent measurement of the SHG (sweep rate 1.5 K/min). The transition temperature was determined by piece-wise fitting of linear functions and by calculating their intersection point. Since the order parameter is proportional to the square root of the SHG intensity, the observed behavior corresponds to the temperature dependence expected for a second-order phase transition.

## 4. Conclusions

The single crystals of LiMnPO_4_ have been grown by the traveling-solvent floating-zone technique under high Ar pressure, and the optical and laser properties such as the absorption spectra and the second harmonic generation (SHG) have been firstly investigated. The grain selection is disabled by a Mn-depleted secondary phase to the end of the growth process. However, good-quality crystals could be analyzed by powder diffraction, a four-circle diffractometer, and physical measurements. The annealing of crystals leads to a change in color but leaves the magnetic behavior virtually unaffected. However, it has no consequence on magnetic data, so we think that it is only a surface effect. The single crystals of Li(Mn*_1-x_*,Ni*_x_*)PO_4_ (0 < *x* < 0.5) have been grown by the traveling-solvent floating-zone technique under high Ar pressure. The polarized microscopic images and Laue patterns prove the excellent quality of the single crystals. The optical and laser properties, such as the absorption spectra and the second harmonic generation, have been firstly investigated. Oriented cuboids with sizes of 2.7 × 3.8 × 2.1 mm^3^ along the *a*, *b*, and *c* crystalline directions were cut and polished. The single-crystal structures were refined by GSAS code, and we found that the *a* and *b* parameters decrease with an increasing Ni concentration, whereas the *c* parameter decreases with an increasing Ni-doping lower than 5%. Two strong absorption peaks are observed as 1.4 and 2.7 eV, corresponding to the *d^8^* orbital of Ni^2+^ and *d^5^* of Mn^2+^ ions, respectively. The lower-energy SHG signal shows a decrease in temperature and vanishes around 32 K, close to the Neel Temperature T_N_ = 32.5 K of pure LiMnPO_4_.

## Figures and Tables

**Table 1:** The chemical compositions collected on the three spots on the (001) surface of the Li(Mn_0.95_,Ni_0.05_)PO_4_ crystal.

Measurement Spots	Element	Wt %	At %	K-Ratio	Z	A	F
1	P*κ*	34.63	48.56	0.2532	1.0667	0.6839	1.0024
	Mn*κ*	60.77	48.04	0.5760	0.9598	0.9824	1.0052
	Ni*κ*	4.60	3.40	0.0424	0.9943	0.9272	1.0000
	Total	100.00	100.00				
1	Mn*κ*	93.72	94.10	0.9399	0.9976	0.9990	1.0062
	Ni*κ*	6.28	5.90	0.0584	1.0348	0.8978	1.0000
	Total	100.00	100.00				
2	Mn*κ*	92.64	93.08	0.9296	0.9972	0.9989	1.0074
	Ni*κ*	7.36	6.92	0.0684	1.0344	0.8990	1.0000
	Total	100.00	100.00				
3	Mn*κ*	92.53	92.97	0.9285	0.9972	0.9988	1.0075
	Ni*κ*	7.47	7.03	0.0695	1.0343	0.8991	1.0000
	Total	100.00	100.00				

## Data Availability

Data Sharing is not applicable.
